# Flexible and Scalable Full‐Length CYP2D6 Long Amplicon PacBio Sequencing

**DOI:** 10.1002/humu.23166

**Published:** 2017-01-18

**Authors:** Henk P.J. Buermans, Rolf H.A.M. Vossen, Seyed Yahya Anvar, William G. Allard, Henk‐Jan Guchelaar, Stefan J. White, Johan T. den Dunnen, Jesse J. Swen, Tahar van der Straaten

**Affiliations:** ^1^ Leiden Genome Technology CenterDepartment of Human Genetics, Leiden University Medical CenterLeiden2333ZCThe Netherlands; ^2^ Department of Clinical Pharmacy & ToxicologyLeiden University Medical CenterLeiden2333ZAThe Netherlands; ^3^ Department of Clinical GeneticsLeiden University Medical CenterLeiden2333ZCThe Netherlands

**Keywords:** pharmacogenomics, *CYP2D6*, variant phasing, copy‐number variation, PacBio long‐read sequencing

## Abstract

Cytochrome P450 2D6 (*CYP2D6*) is among the most important genes involved in drug metabolism. Specific variants are associated with changes in the enzyme's amount and activity. Multiple technologies exist to determine these variants, like the AmpliChip CYP450 test, Taqman qPCR, or Second‐Generation Sequencing, however, sequence homology between cytochrome P450 genes and pseudogene *CYP2D7* impairs reliable *CYP2D6* genotyping, and variant phasing cannot accurately be determined using these assays. To circumvent this, we sequenced *CYP2D6* using the Pacific Biosciences RSII and obtained high‐quality, full‐length, phased *CYP2D6* sequences, enabling accurate variant calling and haplotyping of the entire gene‐locus including exonic, intronic, and upstream and downstream regions. Unphased diplotypes (Roche AmpliChip CYP450 test) were confirmed for 24 of the 25 samples, including gene duplications. Cases with gene deletions required additional specific assays to resolve. In total, 61 unique variants were detected, including variants that had not previously been associated with specific haplotypes. To further aid genomic analysis using standard reference sequences, we have established an LOVD‐powered *CYP2D6* gene‐variant database, and added all reference haplotypes and data reported here. We conclude that our *CYP2D6* genotyping approach produces reliable *CYP2D6* diplotypes and reveals information about additional variants, including phasing and copy‐number variation.

## Introduction

Cytochrome P450 2D6 (*CYP2D6*) is one of the most important genes in pharmacogenetics [Mahgoub et al., 1977; Eichelbaum et al., 1979]. The enzyme metabolizes about 25% of all prescription drugs [Owen et al., 2009] and the *CYP2D6* gene is highly polymorphic, with over 100 genetic variants reported, including copy‐number variation (CNV) and gene rearrangements [Gaedigk, 2013]. Different *CYP2D6* genotyping technologies are available, including Taqman qPCR assays, microarrays, classical Sanger sequencing, and next‐generation sequencing (e.g., exome‐ and whole‐genome sequencing). The AmpliChip CYP450 test from Roche Diagnostics was the first US FDA approved array to genotype *CYP2D6* in a diagnostic setting by profiling a preselected number of variants [Rebsamen et al., 2008]. However, due to the high costs and inability to add novel SNVs to the AmpliChip, this array was discontinued. Multiple alternative platforms exist, such as the xTAG CYP2D6 kit (Luminex), AutoGenomics [Vairavan, 2004], and the Genochip *CYP2D6* [Bank et al., 2015] (Pharmgenomics). Also these assays cannot detect which allele is duplicated, determine the copy number and are unable to detect complex structural variants. Moreover, sequence homology between several *CYP450* genes and pseudogenes *CYP2D7/2D8* impairs reliable *CYP2D6* genotyping, especially for second‐generation sequencing [Drögemöller et al., 2013]. In addition, variant phasing, that is, to identify the linkage of SNVs or haplotypes present in a subject, cannot be accurately determined with these assays.

Targeted long‐amplicon sequencing using the PacBio single‐molecule real‐time (SMRT) sequencing platform offers many advantages over these routinely used assays for genotyping. The main advantage comes from the ability to handle and generate continuous sequence reads from template molecules of multiple kilobases in length without the need for DNA fragmentation steps. The current P6‐C4 polymerase and chemistry release yields average read lengths of 15 kb. Long sequence reads are pivotal to accurately identify and exclude off‐target signals from homologous sequences in the genome of interest, such as pseudogenes. In addition, the PacBio platform has a context‐free error profile, allowing for high‐quality consensus reads of >QV50 (one error in 10^5^ bases) to be generated from the relatively high‐error single‐pass data [Travers et al., 2010; Carneiro et al., 2012]. This combination of long reads and high‐quality sequences allows for accurate variant calling and phasing of multiple heterozygous variants, potentially separated by several kilobases on the genome, into separate haplogroup sequences.

The current output of the PacBio system could accommodate sequencing of multiple samples on a single SMRT cell, depending on the size of the target region of interest. Options for multiplexing are needed in order to make *CYP2D6* genotyping with PacBio cost‐efficient, flexible, and scalable. In this paper, we present a two‐step PCR‐based barcoding scheme for PacBio‐targeted long‐amplicon sequencing. Compared with the recent publication of Qiao et al. (2016) that describes PacBio sequencing for *CYP2D6* haplotyping covering only the coding sequences, our setup targets a larger gene region that also includes the promoter and downstream gene regions. Moreover, the PCR setup we have applied is less laborious compared to that described in Qiao et al. (2016), and our approach is universally applicable due to the use of generic M13 sequences. After applying our method, we found that full‐length *CYP2D6* could be sequenced reliably. We detected 61 unique variants across all samples, while retaining accurate phasing information. With the exception of one sample, the previously established diplotypes by the Roche AmpliChip CYP450 test were confirmed by the PacBio data. We conclude that this approach is cost‐efficient and reveals complete and reliable information about all variants in *CYP2D6*, including phasing and CNVs.

## Methods

### Long‐Range PCR, SMRT Library Prep, and PacBio Sequencing

All work described in this paper is subject to the LUMC Good Research Practice & Integrity guidelines and Ethical requirements. Samples were selected to represent a clinically relevant set of *CYP2D6* haplogroups from the CYPTAM study (The Netherlands Trial Register 1509) and other anonymized samples. *CYP2D6* genotypes were established by Roche AmpliChip CYP450 test (Roche, Almere, The Netherlands) as previously described [Dezentjé et al., 2015]. PCR primers used in this study (Supp. Table S1) were obtained from IDT‐DNA Technologies Leuven, Belgium. All oligos with barcode sequences were ordered as HPLC‐purified. Other primers were ordered as standard desalted. The *CYP2D6‐*specific primer sequences, used to generate a 6.6‐kb fragment covering the *CYP2D6* gene locus including up and downstream regions, were based on Gaedigk et al. (2007). These primers exclude the *CYP2D7* and *CYP2D8* pseudogenes from downstream analysis.

Direct barcoding: sample‐specific barcode sequences were introduced during a single PCR reaction using barcoded fusion primers. The target regions were amplified using the Takara‐v2 kit in a 25‐μl reaction volume containing 400 nM gene‐specific primers, 50–100 ng genomic DNA, 400 mM of each dNTP, 1x PCR buffer with 2.5 mM MgCl2, and 1 U Takara LA Taq. Cycle parameters were 3' at 95°C, followed by 30 cycles of 10'' at 98°C and 15' at 68°C, and a final extension of 15' at 68°C. The length of the products was confirmed on a 1% agarose gel or Bioanalyzer 12000 chip (Agilent Amstelveen, the Netherlands). All samples were pooled in equimolar amounts and the sample pool was purified with 0.5x volume AMPure XP Beads (Beckman‐Coulter Woerden, the Netherlands) and eluted in 30 μl 10 mM Tris–HCl, pH 8.5 prior to PacBio library preparation.

**Figure 1:**
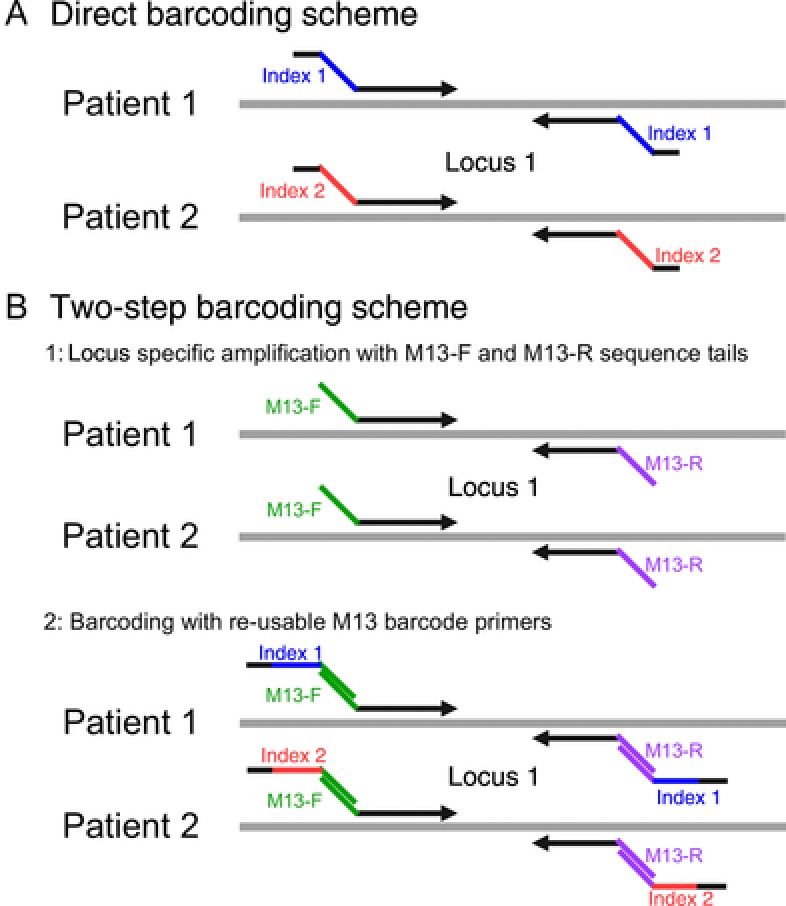
Barcoding schemes. Direct versus two‐step sample barcoding. **A**: In the direct barcoding scheme the sample specific barcodes, indicated by the blue and red for patients 1 and 2, respectively, are attached to the gene‐specific sequences (black arrows) and are introduced in a single PCR reaction. **B**: For the two‐step procedure for each individual, the region of interest is first amplified with a pair of gene‐specific primers with M13 forward (green) and reverse (purple) sequence tails. A symmetrical sample barcode, indicated by blue and red for patients 1 and 2, respectively, is introduced in a second PCR using a set of M13 barcode primers. A 5' padding sequence (black) is present on the index primers for both the direct and two‐step barcoding schemes to give all fragment identical end sequences to avoid ligation biases during the SMRT bell library preparation.

Two‐step barcoding: the *CYP2D6*‐specific amplification, QC, and pooling were performed with M13‐tailed primers using identical conditions as the PCR of the direct barcoding scheme. Sample‐specific barcodes were introduced in a second PCR with identical conditions as the first, but using 3 μl of the purified product from PCR #1 and #5 cycles of amplification. The direct and two‐step barcode schemes are summarized in Figure 1A and B, respectively.

Sequence libraries were prepared from the pooled amplicons following the standard procedures for SMRT‐loop adapter ligation, starting from 500 ng of the pooled fragments. Sequencing of the libraries was performed with standard procedures using either the P4 or P6 enzyme.

### PCR Detection for *CYP2D6* Gene Deletion, Gene Duplication, and *CYP2D6‐7* Fusion Genes

CYP2D6^*^5 gene deletion events were detected using a duplex PCR assay [Gaedigk et al., 2008], and *CYP2D6* gene duplications and *CYP2D6‐7* fusion events were assayed by a triplex PCR [Gaedigk et al., 2007; Gaedigk and Coetsee, 2008] using KAPA LongRange HotStart reagents. Reactions (25 μl) contained 1x reaction buffer, 1.75 mM MgCl2, 0.3 mM dNTP (each), 0.625 U KAPA LongRange HotStart DNA Polymerase, 10 ng human genomic DNA, and 1.25 μl of primer mix (Supp. Table S1). Triplex reactions were supplemented with 5% DMSO. Cycle parameters were 3' at 95°C, followed by 35 cycles of 15'' at 95°C and 10' at 68°C, with a final extension of 15' at 68°C. PCR products were visualized using a Genomic DNA ScreenTape assay on the Agilent 4200 TapeStation system.

### PacBio Sequence Data Processing

For the *CYP2D6* data, phased haplogroup sequences were retrieved for each individual using the long‐amplicon analysis (v2) protocol in the PacBio SMRT portal (v2.3.0). Analysis settings for both the two‐step barcoding and direct barcoding setup were: minimum subread length = 6,000; maximum number of subreads = 800; ignore primer sequence = 21; trim ends = 21; only most supported = 0; cluster per gene fam = y; phase alleles = y; split results = n; MinSnr = 4.5. The resulting haplogroup sequences were manually inspected on length and subread coverage to exclude spurious artefact sequences. All sequences were orientated to the plus strand and gene‐specific and M13 primer sequences were removed (cutadapt v1.7.1) [Martin, 2011] prior to aligning the reads to chr22 of the human genome (GRCh38) with BWA MEM (v1.7.1) [Li et al., 2009]. Bam and pileup files were generated using Samtools (v1.2) [Li et al., 2009; Li, 2011], and variants were called with bcftools (v1.2; bcftools call ‐mv ‐Ov ‐P 0.99 ‐p 0.99 | bcftools norm ‐m ‐both). All variants were merged into one vcf file, and described in HGVS format. A fasta file with all full‐length *CYP2D6* sequences is available in Supp. Information. All variants have been submitted to the LOVD [Fokkema et al., 2011] *CYP2D6* database (www.LOVD.nl/CYP2D6).

### *CYP2D6* Genotype Calling

The translation table listing the allele to genotype information was obtained from the Pharmacogenomics Knowledgebase (PharmGKB) [Whirl‐Carrillo et al., 2012] (http://www.pharmgkb.org/; version February 4, 2016). All variants were lifted from the M33388 format to GRCh38 genomic positions in HGVS format and checked with Mutalyzer v2.0.7 [Wildeman et al., 2008]. For future use and to support variant descriptions using different reference sequences, all *CYP2D6* reference haplotypes were submitted to the LOVD database (http://www.LOVD.nl/CYP2D6). The haplogroup sequences per individual were processed separately to assign genotype by matching the individual variants to genotypes via the PharmGKB translation table.

## Results

### Full‐Length *CYP2D6* Sequencing Using Direct Barcoding

**Figure 2:**
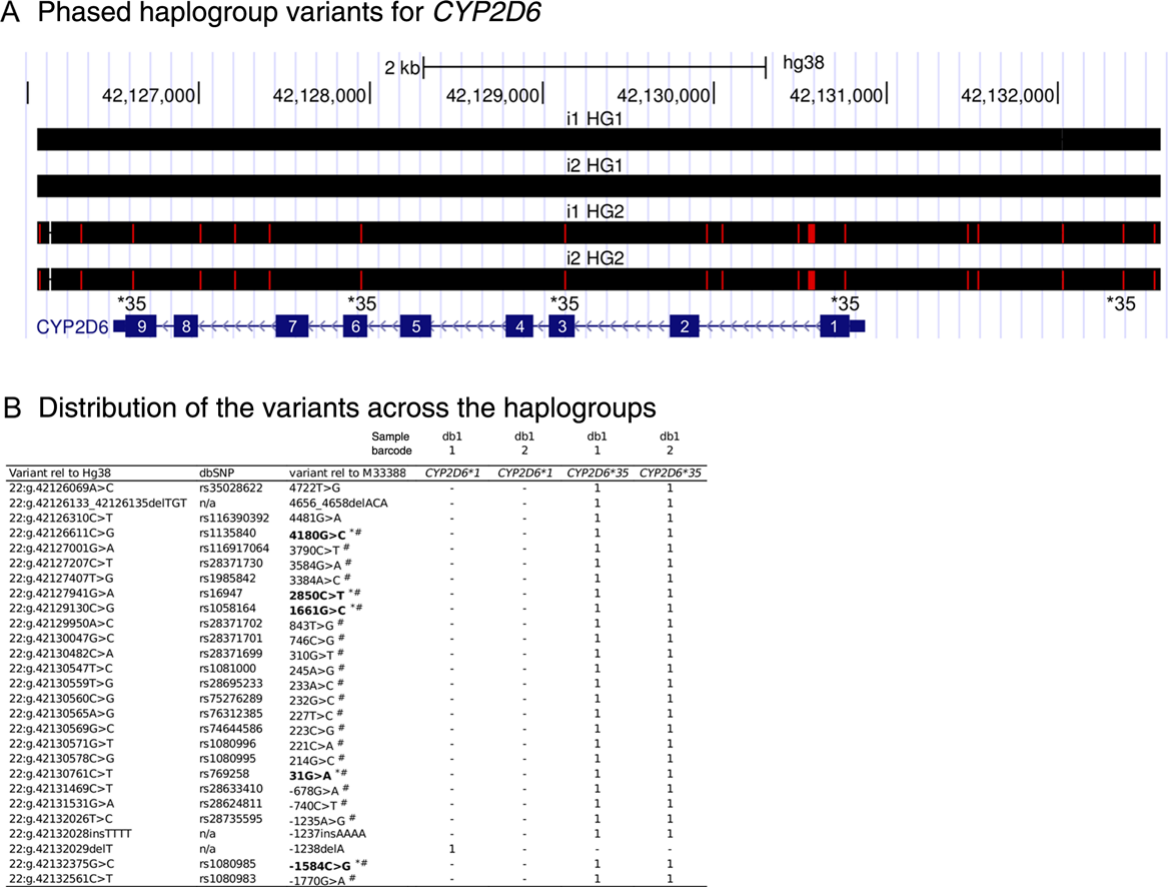
Direct barcoding results. Direct barcoding results for the technical replicate with different barcodes. **A**: UCSC browser screenshot illustrating the detected variants (red lines) of the two fully‐phased haplogroup sequences for each sample barcode relative to the GRCh38 reference. The *CYP2D6* gene is located on the negative strand. Exon numbers are indicated in white based on the NM_000106.5 transcript sequence. For the haplogroup 2 sequences, the variants determining the CYP2D6^*^35 call are indicated. **B**: Identity and distribution of all 27 variants across the haplogroup sequences. Order in this table (top to bottom) is identical to those in Figure 2A (left to right). Variants in bold determine the CYP2D6^*^35 haplotype; ^*^ indicates that the variant was included on the Roche AmpliChip CYP450 test; # indicates that the variant was included in PharmGKB (February 4, 2016); and “HG” and “i” denote haplogroup and index, respectively.

Barcoded fusion primers for amplification of full‐length *CYP2D6* were designed based on the PacBio multiplex PCR primer guidelines. Initial analysis of one individual was performed using two technical replicate libraries with unique barcodes, prepared and sequenced together on one PacBio SMRT cell. Full‐length *CYP2D6* sequences resulted after barcode demultiplexing and processing the data with the Long‐Amplicon Analysis software. Two different sequences with approximately equal subread coverage (226 vs. 200 reads) were identified for this individual, indicating the presence of two distinct *CYP2D6* haplogroup sequences (Table 1; Supp. Table S2). No chimeric long‐range PCR fragments were evident in the data. Variant calling resulted in 24 single‐nucleotide substitutions, one insertion, and two deletions, all of which were heterozygous with one of the deletions located on one haplogroup and the remaining variants located within the other haplogroup (Fig. 2A and B). Genotype calling for the two separate haplogroup sequences indicated the CYP2D6^*^1/^*^35A diplotype for this individual (CYP2D6^*^35A was based on variant rs769258 and s1135840; rs16947; rs1058164; rs1080985; Fig. 2B). It should be noted that since the entire gene was sequenced, including intronic and direct flanking regions, we generated a complete haplotype including a range of variants that have so far not been reported to be associated with the CYP2D6^*^35 haplotype. CYP2D6^*^1/^*^35A predicts a normal metabolizer phenotype, consistent with the AmpliChip array result. The technical replicate was in full agreement with haplogroup 2 for barcode 1 except for an one nucleotide length difference in a 22‐bp T‐homopolymer located in the upstream region of *CYP2D6* (22:g.42132029delT) for haplogroup 2. Different settings for the long‐amplicon analysis could not resolve this discrepancy.

**Table 1:** PacBio Haplogroup and Genotype Data

Sample	Haplo group	Coverage ratio	SUB–INS–DEL	Call het/hom	PacBio‐based genotype	PacBio metabolizer group	AmpliChip CYP450 test genotype	AmpliChip metabolizer group
db1 index1	1	1:1	0–0–1	Het 27/hom 0	CYP2D6 ^*^1	Normal	CYP2D6 ^*^1/^*^35	Normal
	2	1	24–1–1		CYP2D6 ^*^35A			
db1 index2	1	1:3	0–0–0	Het 26/hom 0	CYP2D6 ^*^1	Normal	CYP2D6 ^*^1/^*^35	Normal
	2	1	24–1–1		CYP2D6 ^*^35A			
1	1	1	0–0–1	het 28/hom 0	CYP2D6 ^*^1	Normal	CYP2D6 ^*^1/^*^35	Normal
	2	1:3	25–1–1		CYP2D6 ^*^35A			
2	1	1:1	0–0–0	het 25/hom 0	CYP2D6 ^*^1	Normal	CYP2D6 ^*^1/^*^2	Normal
	2	1	23–1–1		CYP2D6 ^*^2A			
3	1	1	1–0–0	het 27/hom 0	CYP2D6 ^*^1	Normal	CYP2D6 ^*^1/^*^2	Normal
	2	1:3	24–1–1		CYP2D6 ^*^2A			
4	1	1	1–0–1	het 27/hom 0	CYP2D6 ^*^1B	Ultrarapid	CYP2D6 ^*^1/^*^2XN	Ultrarapid
	2	2:3	23–1–1		CYP2D6 ^*^2AXN			
5	1	1	1–0–0	het 26/hom 0	CYP2D6 ^*^1	Intermediate	CYP2D6 ^*^1/^*^41	Intermediate
	2	1:0	23–1–1		CYP2D6 ^*^41			
6	1	1	0–1–0	het 3/hom 0	CYP2D6 ^*^1	Normal	CYP2D6 ^*^1/^*^1	Normal
	2	1:0	1–0–1		CYP2D6 ^*^1			
7	1	1	0–0–1	het 27/hom 0	CYP2D6 ^*^1	Normal	CYP2D6 ^*^1/^*^2	Normal
	2	1:2	24–1–1		CYP2D6 ^*^2A			
8	1	1	19–1–0	het 30/hom 8	CYP2D6 ^*^4A	Intermediate	CYP2D6 ^*^4/^*^35	Intermediate
	2	1:1	24–1–1		CYP2D6 ^*^35A			
9	1	1	18–1–0	het 28/hom 8	CYP2D6 ^*^4A	Intermediate	CYP2D6 ^*^2/^*^4	Intermediate
	2	1:0	23–1–1		CYP2D6 ^*^2A			
10	1	1:1	23–1–1	het 5/hom 23	CYP2D6 ^*^41	Intermediate	CYP2D6 ^*^2/^*^41	Intermediate
	2	1	24–1–1		CYP2D6 ^*^2A			
11	1	1:0	1–0–1	het 27/hom 0	CYP2D6 ^*^1	Intermediate	CYP2D6 ^*^1/^*^41	Intermediate
	2	1	23–1–1		CYP2D6 ^*^41			
12	1	5:6	18–1–0	het 0/hom 19	CYP2D6 ^*^4A/^*^5	Poor	CYP2D6 ^*^4/^*^4	Poor
13	1	1	0–0–2	het 27/hom 0	CYP2D6 ^*^3A	Intermediate	CYP2D6 ^*^2/^*^3	Intermediate
	2	1:0	23–1–1		CYP2D6 ^*^2A			
14	1	1	24–1–1	het 0/hom 26	CYP2D6 ^*^5/^*^35A	Intermediate	CYP2D6 ^*^5/^*^35A	Intermediate
15	1	1	14–1–0	het 0/hom 15	CYP2D6 ^*^10D/^*^10D	Intermediate	CYP2D6 ^*^10D/^*^10D	Intermediate
16	1	1	2–0–2	het 30/hom 0	CYP2D6 ^*^9	Intermediate	CYP2D6 ^*^9/^*^35	Intermediate
	2	1:7	24–1–1		CYP2D6 ^*^35A			
17	1	1:4	2–0–1	het 22/hom 0	CYP2D6 ^*^6B	Poor	CYP2D6 ^*^4/^*^6	Poor
	2	1	18–1–0		CYP2D6 ^*^4A			
18	1	1	2–0–2	het 23/hom 1	CYP2D6 ^*^1	Intermediate	CYP2D6 ^*^1/^*^17	Intermediate
	2	1:2	20–1–0		CYP2D6 ^*^17			
19	1	1	23–1–1	het 0/hom 25	CYP2D6 ^*^2A/^*^5	Intermediate	CYP2D6 ^*^2/^*^5	Intermediate
20	1	1	23–1–1	het 0/hom 25	CYP2D6 ^*^2A/^*^2AXN	Ultrarapid	CYP2D6 ^*^2/^*^2XN	Ultrarapid
21	1	1	0–0–1	het 26/hom 0	CYP2D6 ^*^9	Intermediate	CYP2D6 ^*^9/^*^41XN	Intermediate
	2	2:1	23–1–1		CYP2D6 ^*^41XN			
22	1	2:2	1–0–1	het 28/hom 0	CYP2D6 ^*^1XN	Ultrarapid	CYP2D6 ^*^1XN/^*^35	Ultrarapid
	2	1	24–1–1		CYP2D6 ^*^35A			
23	1	1:2	1–0–1	het 21/hom 0	CYP2D6 ^*^1	Intermediate	CYP2D6 ^*^1/^*^4	Intermediate
	2	1	18–1–0		CYP2D6 ^*^4A			
24	1	1	1–0–2	het 22/hom 0	CYP2D6 ^*^1	Intermediate	CYP2D6 ^*^1/^*^4	Intermediate
	2	1:1	18–1–0		CYP2D6 ^*^4A			

Summary of the *CYP2D6* genotyping data describing the number of variants found for the separate haplogroup sequences for each sample. Indicated from left to right for the duplicate direct barcoded sample and each of the 24 two‐step barcoded samples are the haplogroup number; coverage ratio between haplogroups; the number of single‐nucleotide substitutions, insertions, and deletions (SUB—INS–DEL) per haplogroup; and the number of heterozygous and homozygous variants. The last four columns describe the *CYP2D6* genotype and the predicted metabolizer group derived from the PacBio data and RocheAmpliChip CYP450 test.

### M13 Sequence‐Based Two‐Step Barcoding Scheme For Multiplexing of *CYP2D6*

Although the direct barcoding scheme is able to deliver high‐quality results and accurate *CYP2D6* phenotype predictions, the setup is rigid. Extending the existing setup to cover more targets would require a complete set of new barcoded fusion primer pairs for each additional target. In parallel, for each additional individual to be sequenced on the same SMRT cell, two additional, expensive, HPLC‐purified barcoded primers for each target in the experiment design are needed. Therefore, we set up a generally applicable, versatile, and cost‐efficient PCR‐based multiplexing strategy for long‐amplicon sequencing based on a two‐step barcoding system. In this scheme, the ∼6.6‐kb *CYP2D6* gene locus, including the upstream and downstream regions, is first amplified with a pair of gene‐specific primers with forward and reverse M13 sequence tails for each individual sample separately. A symmetrical sample barcode is subsequently introduced in a second PCR using a set of generic M13‐tailed barcode primers.

Using this setup, we sequenced *CYP2D6* for 24 individuals with different predicted *CYP2D6* phenotypes based on the Roche AmpliChip CYP450 test assay representing the *CYP2D6* ^*^1‐6, ^*^9, ^*^10, ^*^17, ^*^35, and ^*^41 haplogroups. Samples received a unique barcode during the second round of PCR, and all barcodes could be identified in the data with approximately equal numbers of subread coverage in a multiplex of 12 samples per SMRT cell (Supp. Table S2). Full‐length *CYP2D6* sequences were generated for all samples without evidence for chimeric sequences. For five individuals, a single haplogroup sequence was obtained. For the remaining samples, two separate haplogroup sequences were found, 15 of which contained only heterozygous variants across the two haplogroups. The other four individuals showed the presence of both heterozygous and homozygous variants (Table 1).

In total, 695 variants were detected for the 24 individuals, representing 61 unique variants, comprising 49 single‐nucleotide substitutions, five insertions, and seven deletions. The majority of the variants reside in the noncoding regions of the *CYP2D6* gene: 18 (30%) were upstream, 20 (33%) intronic, and five (8%) downstream gene variants. Supp. Figure S1 shows the distribution of these variants across the *CYP2D6* locus, alongside the variants on the Roche AmpliChip CYP450 test and those included in PharmGKB. Eighteen variants in the coding regions represent 10 missense mutations, two frameshift mutations, one in‐frame deletion, and one splice‐acceptor variant, with the remaining four being synonymous variants. Of the 61 unique variants, nine have not previously been described in dbSNP. Of these, seven are associated with a long T‐homopolymer stretch, leaving one novel deletion (22:g.42126133_42126135delTGT), which was present in 17 of the 24 individuals in this study in the downstream region of *CYP2D6* gene region, and one SNV (22:g.42131610G>C) present in one sample in the upstream gene region. Sanger sequencing confirmed both novel variants (Supp. Fig. S2).

Phenotype predictions for these individuals for the separate haplogroup sequences were all in agreement with those obtained from the AmpliChip (Table 1). Moreover, using the PacBio data, we were able to more specifically type a subset of the haplogroup calls. One of the CYP2D6^*^1 haplogroups could now be called specifically as CYP2D6^*^1B based on the presence of the 22:g.42126963C>T variant. Several samples were called to have the CYP2D6^*^4ABDJK haplogroup by the Roche AmpliChip CYP450 test based on the 1846G>A (rs3892097) variant, whereas the PacBio was able to refine the call to CYP2D6^*^4A, based on all seven variants for this haplogroup. Similarly, CYP2D6^*^2ABD haplotypes, based on two variants, could now be matched to CYP2D6^*^2A; based on 14 variants, CYP2D6^*^35 haplogroups could more precisely be defined as CYP2D6^*^35A and CYP2D6^*^6 as CYP2D6^*^6A.

In total, 19 variants were not present in PharmGKB, meaning data linking these to specific haplogroups is currently lacking. Nine of these variants were found in more than one haplogroup, and 10 variants were unique to specific haplotypes. The downstream gene variant 22:g.42126079C > T was only present in the CYP2D6^*^17 haplogroup, the intronic 22:g.42129545G > A variant only in CYP2D6^*^6B, and the two upstream gene variants, 22:g.42131610G > C and 22:g.42131631A > T, were both present in one of the CYP2D6^*^9 haplotypes. Also, two intronic and one upstream gene variant were detected in CYP2D6^*^1 haplotypes only, two of which, 22:g.42129623C > T and 22:g.42130522G > A, had previously also been identified by Twist et al. (2016). One of the CYP2D6^*^35 haplogroup samples contained the 22:g.42126944C > T missense variant, but PolyPhen [Adzhubei et al., 2010], SIFT [Kumar et al., 2009], and Condel [González‐Pérez and López‐Bigas, 2011] scored this as “tolerated,” “benign,” and “neutral,” respectively. Two of the variants not listed in PharmGKB were found in CYP2D6^*^2A haplotypes, with one located in the upstream gene region (22:g.42131114A > G) and one missense variant (22:g.42127611C > T), with “deleterious” SIFT and Condel scores and “benign” according to PolyPhen.

The PacBio data were able to identify CNVs for samples that carry at least two distinctive *CYP2D6* haplotype sequences. For samples 4, 21, and 22, an unequal subread coverage for the distinct haplogroups was observed, indicating a duplication of one of the haplogroups. For instance, in sample 4, the ratio of reads for the *CYP2D6* ^*^ *2A* and CYP2D6^*^1B diplotype was 2.3 to 1, indicating a duplication of the ^*^2A haplogroup sequence for this individual, which was consistent with the results derived from the AmpliChip CYP450 test as well as the Triplex PCR fragment to detect *CYP2D6* gene duplication events (Supp. Fig. S3). For sample 20, a single CYP2D6^*^2A haplotype was observed, and the Triplex PCR indicated a gene duplication, supporting a duplication of the CYP2D6^*^2A haplogroup in combination with a single CYP2D6^*^2A haplogroup on the other chromosome, indicating a CYP2D6^*^2A/^*^2AXN diplotype.

Four other samples (12, 14, 15, and 19) resulted in a single *CYP2D6* haplogroup sequence. The Duplex PCR assay detected CYP2D6^*^5 gene deletions for 12, 14, and 19 (Supp. Fig. S4), indicating the PacBio sequence represents the gene copy of one chromosome only. For sample 12, this gene deletion was not detected with the AmpliChip CYP450 test, which reported a CYP2D6^*^4/^*^4 diplotype instead of CYP2D6^*^4/^*^5, making this the only discordant call between the two assays. The negative call for a CYP2D6^*^5 gene deletion for sample 15 implies it carries two identical CYP2D6^*^10D haplogroup sequences.

These data indicate that the two‐step barcoding scheme can successfully be used for sample multiplex barcoding and sequencing of multiple long‐range PCR amplicons on a single SMRT cell, generating high‐quality phased variant data that can successfully be used for reliable *CYP2D6* genotyping.

## Discussion

In this paper, we present a reliable method for full‐length sequencing of the 6.6‐kb *CYP2D6* gene, an important pharmacogenetic gene. We describe a two‐step sample barcoding scheme for long‐amplicon sequencing on the PacBio RSII to determine the haplogroup sequences and diplotype. Sample‐specific barcodes are introduced via M13 forward and reverse sequences, which are widely used in both research and clinical genetics laboratories and have previously been used in NGS for adding sample‐specific barcodes [De Leeneer et al., 2011]. Therefore, use of the M13 sequences will facilitate the implementation of PacBio for (long) amplicon sequencing in place of standard Sanger sequencing. In contrast to the direct barcoding with fusion primers, our setup with generic M13 barcodes is scalable and flexible and can easily be adapted to profiling other genomic targets without the need for significant additional investments. For example, extending the existing setup by two target loci for 12 individuals can be achieved by designing just four additional target‐specific M13 tailed, standard desalted, primers. To achieve the same for the direct barcoding method would require an additional 48, expensive, HPLC‐purified fusion primers. The generic use of the barcode primers makes the setup cost‐efficient, flexible, and easy to implement for a wide range of targets with minimal optimization time. Compared with the RocheAmpliChip CYP450 test and the Pharmgenomics Genochip CYP2D6, the PacBio setup for *CYP2D6* genotyping results in an approximately 90% and 20% cost reduction per sample, respectively (reagents only, excluding laboratory utilities, equipment depreciation, and labor for data analysis and wet‐lab). The cost per sample will decrease even more with the higher output of the PacBio Sequel system, making (long)amplicon sequencing on the PacBio a suitable alternative to Sanger and targeted short‐read approaches.

PacBio long‐amplicon sequencing can overcome several of the shortcomings of existing *CYP2D6* genotyping methodologies. The ability to handle and sequence long DNA fragments makes it possible to move from a preselected set of variants or only the exonic regions to the entire 6.6‐kb *CYP2D6* locus, including the promoter region, all introns, and the downstream gene region. Long reads also aid in discriminating the locus of interest from potential off‐target sequences such as the *CYP2D7* pseudogene. This can be achieved at the level of primer design during experiment setup by the larger pool of unique primer sequences in a larger sequence space, as well as during data analysis based on sequence identity. For the *CYP2D6* experiments, no high‐identity off‐target sequence alignments were observed, indicating pseudogene contamination was not present in our data. In addition, the long‐sequence reads provide the opportunity for high‐quality variant phasing, that is, to identify the exact distribution of multiple heterozygous variants on the two separate *CYP2D6* haplogroups across multi‐kb regions that is especially of importance for low‐polymorphic samples. Our data showed clear *CYP2D6* haplogroup sequence separation based on a range of 3–30 heterozygous variants allowing us to make genotype calls on the separate individual haplogroup sequences.

Recently, two papers have described the use of long sequence read NGS platforms for *CYP2D6* genotyping. Using the MinION platform, Ammar et al. (2015) sequenced a 5‐kb PCR amplicon without any sample multiplexing options. The high‐error rate of the MinION system, leading to high number of false‐positive variants, is the main limitation of this approach. Qiao et al. (2016) first performed separate amplification of a “downstream” and a “upstream” fragment, with the latter being used for assessing *CYP2D6* CNVs by gel analysis. Both amplicons were used in a nested PCR and barcoding PCR reaction prior to PacBio RSII sequencing, bringing the total number of PCRs to three. Although successful in identifying the *CYP2D6* diplotypes, the study was also limited to a ∼5‐kb sequence fragment covering only the coding sequences of the *CYP2D6* gene, potentially missing variants affecting regulatory sequence features located in the upstream or downstream gene regions, for example, the promoter region. In our study, we had identified and confirmed the existence of two novel variants in these regions, indicating the added value of including the upstream and downstream gene regions. A main difference between the PacBio studies is that Qiao et al. (2016) corrected PacBio errors based on alignments to a predefined reference, whereas we used the PacBio Long Amplicon Analysis tool, which is an assumption‐free approach, that is, it is independent of any predefined reference sequence. The Long Amplicon analysis is therefore more appropriate for analyzing complex structural rearrangements that would be difficult to align to an existing predefined reference.

Translating a *CYP2D6* genotype to a CYP2D6 phenotype is notoriously difficult, and there is currently no standardized process [Hicks et al., 2014]. Although all PacBio‐derived genotypes were in agreement with those obtained from the RocheAmpliChip CYP450 test, many additional variants were detected in the PacBio data, only a subset (*n* = 42) of which had previously been associated with specific genotypes as described in the PharmGKB [Whirl‐Carrillo et al., 2012] (www.pharmgkb.org; version February 4, 2016). This leaves 19 variants for which the contribution to the *CYP2D6* phenotype is currently unresolved. Some of these variants may potentially help to explain the large interindividual variation in CYP2D6 metabolic capacity typically observed within *CYP2D6* phenotype groups [Schenk et al., 2007]. However, the majority of these variants resided in intronic or either the upstream and downstream gene regions or were missense variants without consequence according to SIFT, PolyPhen, and Condel scores. One missense variant in a CYP2D6^*^2A haplogroup (22:g.42127611C>T; 3180G>A; rs78209835; NP_000097.3:p.Asp337Asn) had SIFT and Condel scores indicating a deleterious effect on protein function, whereas its PolyPhen scored a “benign” effect. Unfortunately, we do not have CYP2D6 protein activity data for this individual, making it unclear whether the variant indeed affects protein function. Further research is needed to determine the exact contribution of these variants.

In addition to these challenges in predicting a CYP2D6 phenotype from a *CYP2D6* genotype, an additional level of complexity lies at the in silico prediction of the variant effects. Different tools are available; however, depending on the settings of these analyses, concordance between these tools may be low [McCarthy et al., 2014]. Also, these prediction tools are based on different sets of assumptions and therefore may produce conflicting predictions for the same variant. In addition, the choice of the reference sequence may affect the prediction results. Prediction tools based on the GRCh19 genome release, representing a CYP2D6^*^2 haplogroup, may have a different sequence context of immediate adjacent bases for a specific variant compared with the GRCh38 release, representing a CYP2D6^*^1 sequence, potentially leading to erroneous conclusions on the effect of a variant. Finally, most tools predict the effect of individual variants, ignoring potential cumulative effects of multiple phased variants across the entire gene locus of the separate haplotypes.

Although the PacBio approach for *CYP2D6* variant phasing and haplotyping was successful, we are aware that a relatively low number of samples were profiled and that these were selected from a mainly Caucasian collection, potentially excluding relevant haplotypes from non‐European descent. Also, no samples with a *CYP2D6‐7* fusion gene were included in this study. An in‐depth analysis of the variants' effects on *CYP2D6* mRNA splicing are not included as these require extensive confirmation of the in silico predictions at the RNA level.

### Sequencing Software Generated Variant Calls in Reference to Genome Build GRCh38

To match the variant calls with *CYP2D6* haplotypes, we checked the Website of the Human Cytochrome P450 (CYP) Allele Nomenclature Committee [Sim and Ingelman‐Sundberg, 2010, 2013] (http://www.cypalleles.ki.se). Unfortunately, variants reported in the *CYP2D6* haplotype table do not follow existing HGVS standards [Den Dunnen and Antonarakis, 2000] (www.HGVS.org/mutnomen). Helpful but indirect links for some variants are given to dbSNP, but overall the variants cannot be used easily. Similarly, the haplotype tables provided by PharmGKB [Whirl‐Carrillo et al., 2012] (www.pharmgkb.org) and the SuperCYP Cytochrome P450 database [Preissner et al., 2010] (http://bioinformatics.charite.de/supercyp/) suffer from the same problem, where nonstandard variant description is used. We therefore decided to add all *CYP2D6* reference haplotypes, using standardized variant descriptions, to the LOVD‐powered *CYP2D6* gene variant database (www.LOVD.nl/CYP2D6). In addition, in collaboration with an international workgroup [Kalman et al., 2016], we generated an upgraded *CYP2D6* haplotype table reporting all variants in relation to all commonly used *CYP2D6* reference sequences (including genome builds GRCh19 and GRCh38, RefSeqGene record NG_008376.3, LRG_303, and reference transcript NM_000106.4). Finally, we submitted all *CYP2D6* data reported here to the *CYP2D6*database. As we sequenced the entire genomic gene segment, including introns and direct gene flanking regions, our data extended several alleles with variants hitherto un‐reported as being part of these alleles.

In summary, using the two‐step barcoding approach, we show that multiplex sequencing of 12 samples for full‐length *CYP2D6* generated reliable sequence information. This approach is cost‐efficient and could represent an improved alternative for existing *CYP2D6* diplotyping technologies for both clinical‐level genotyping and research purposes.

*Disclosure statement*: The authors have no conflict of interest to declare.

## Supporting information

Supporting Information

Supporting Information

Supporting Information

Supporting Information
