# Supplementary material for: Flexible and Scalable Full‐Length CYP2D6 Long Amplicon PacBio Sequencing
Source: Hum Mutat. 2017 Jan 18;38(3):310–6. doi: 10.1002/humu.23166 (PMC5324676; doi:10.1002/humu.23166)
Supplement: Supplementary file 1 — Supporting Information [file HUMU-38-310-s001.doc]

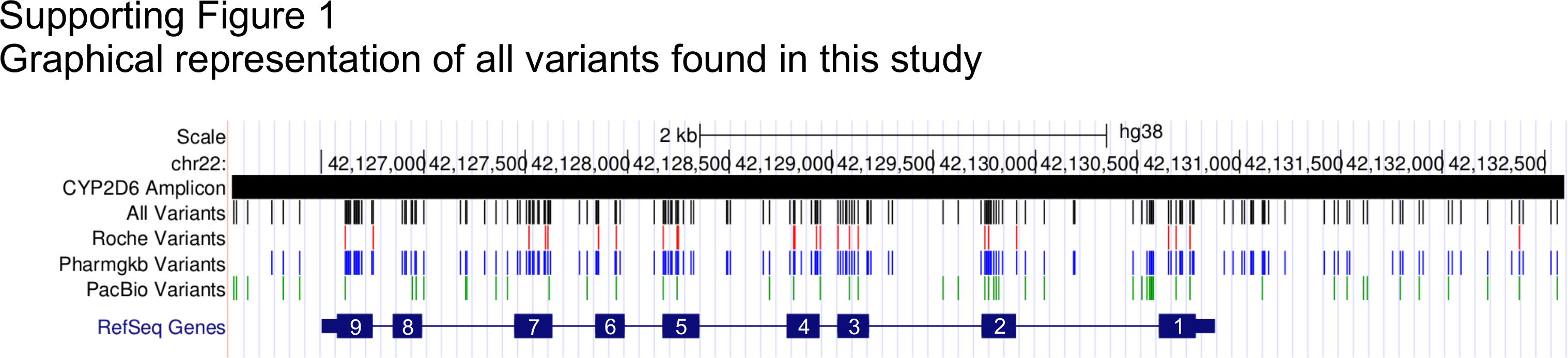


Supporting Figure S1: Visualisation of all variants across the 6.6 kb *CYP2D6* amplicon

USCS screenshot visualising the positions of the variants present in the PacBio data (green), AmpliChip CYP450 test (red), Pharmacogenomics Knowledgebase (blue) in separate tracks in relation to the 6.6 kb amplicon (black bar) that was used for *CYP2D6* haplotyping. The black lines represent all unique variants from these three different sources.


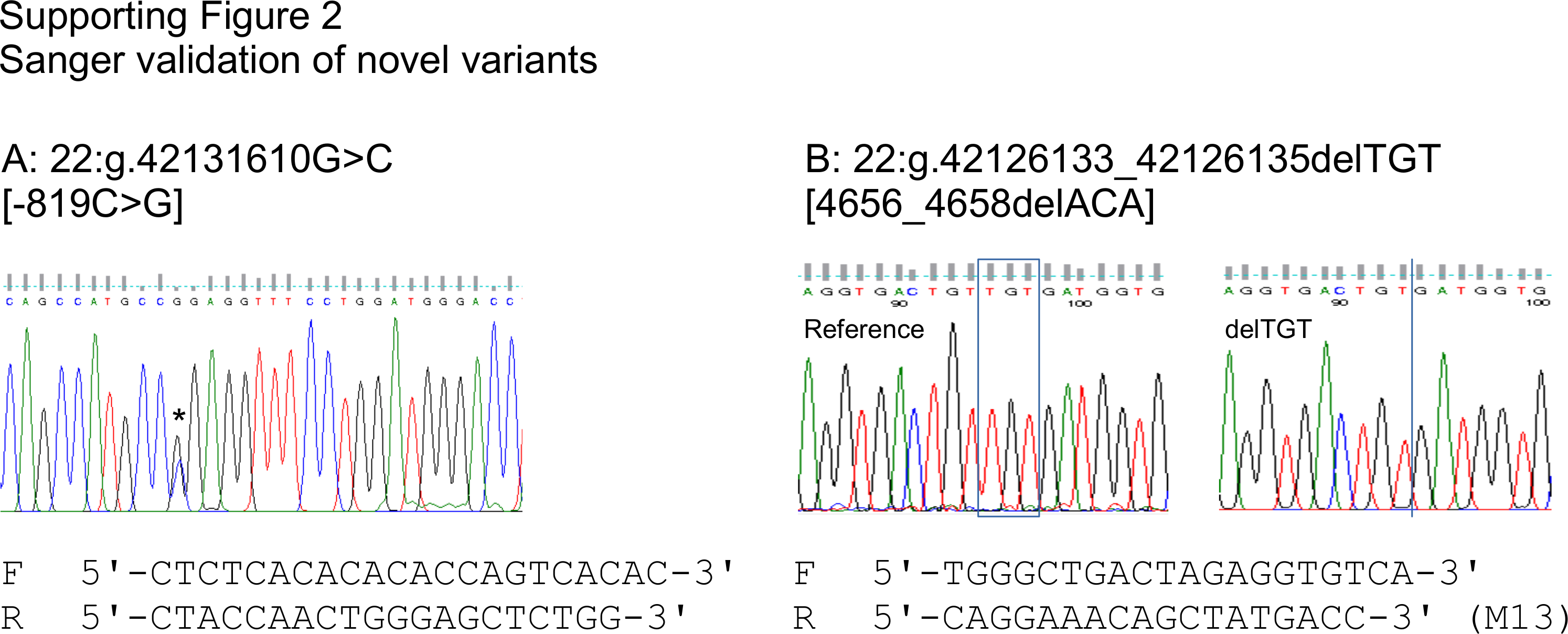


Supporting Figure S2: Validation of novel variants.

Sanger sequencing was performed on nested PCR products from the 6.6 kb *CYP2D6* fragments. For the TGT deletion, the forward primer targets the *CYP2D6* gene, while the Reverse primer that was used is the M13R sequence. All other primers were gene specific.

Supporting Figure S3: *CYP2D6* triplex PCR assays


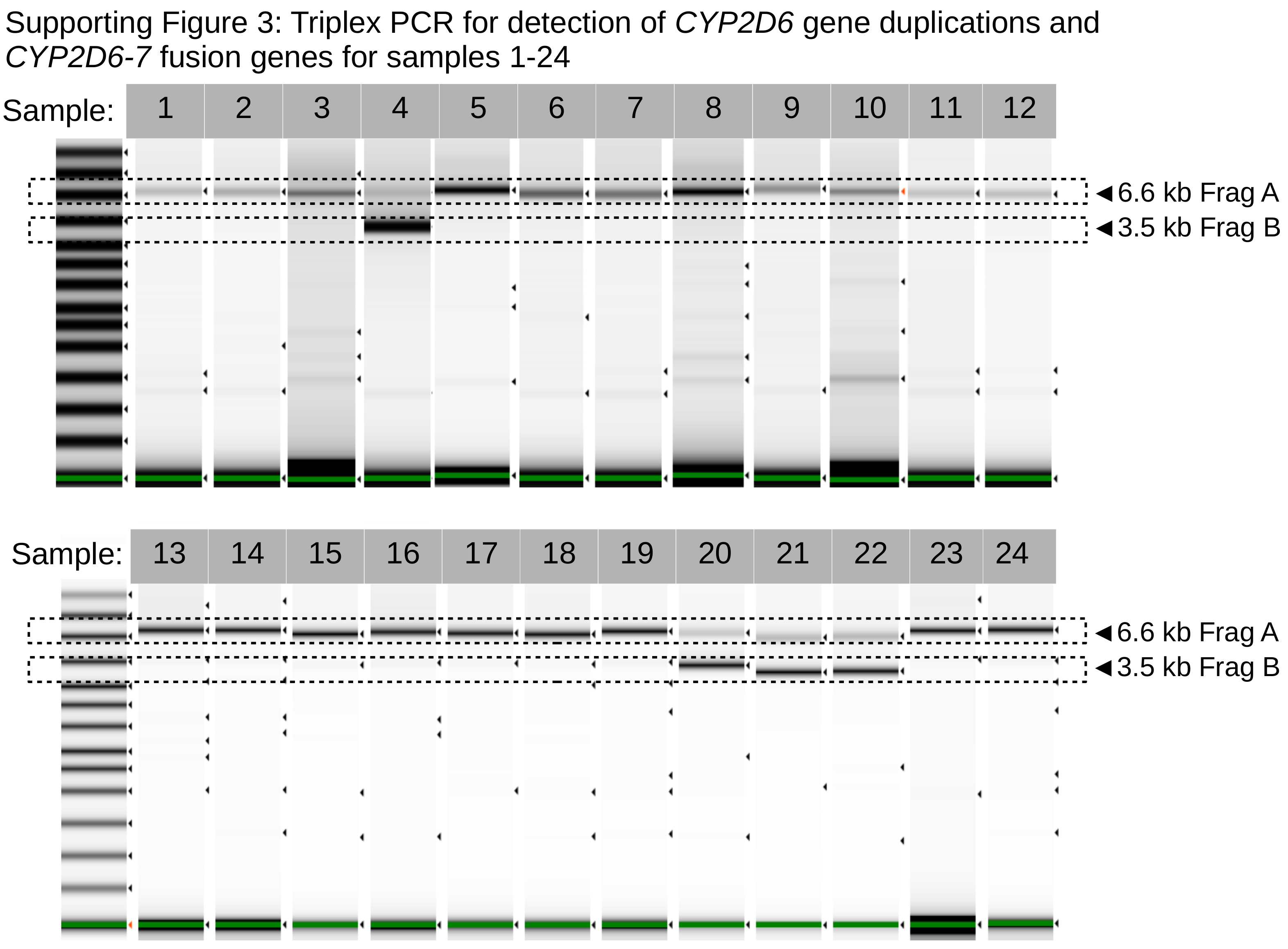
Triplex PCR results for all 24 samples. The 6.6 kb fragment A serves as an internal control and should be present in all reactions. Presence of Fragment B (3.5 kb) is indicative of a *CYP2D6* gene duplication (samples 4,20-22). Samples with *CYP2D6-7* fusion gene events can be identified by presence of Fragment H; 5.0 kb, which was not detected in these samples.


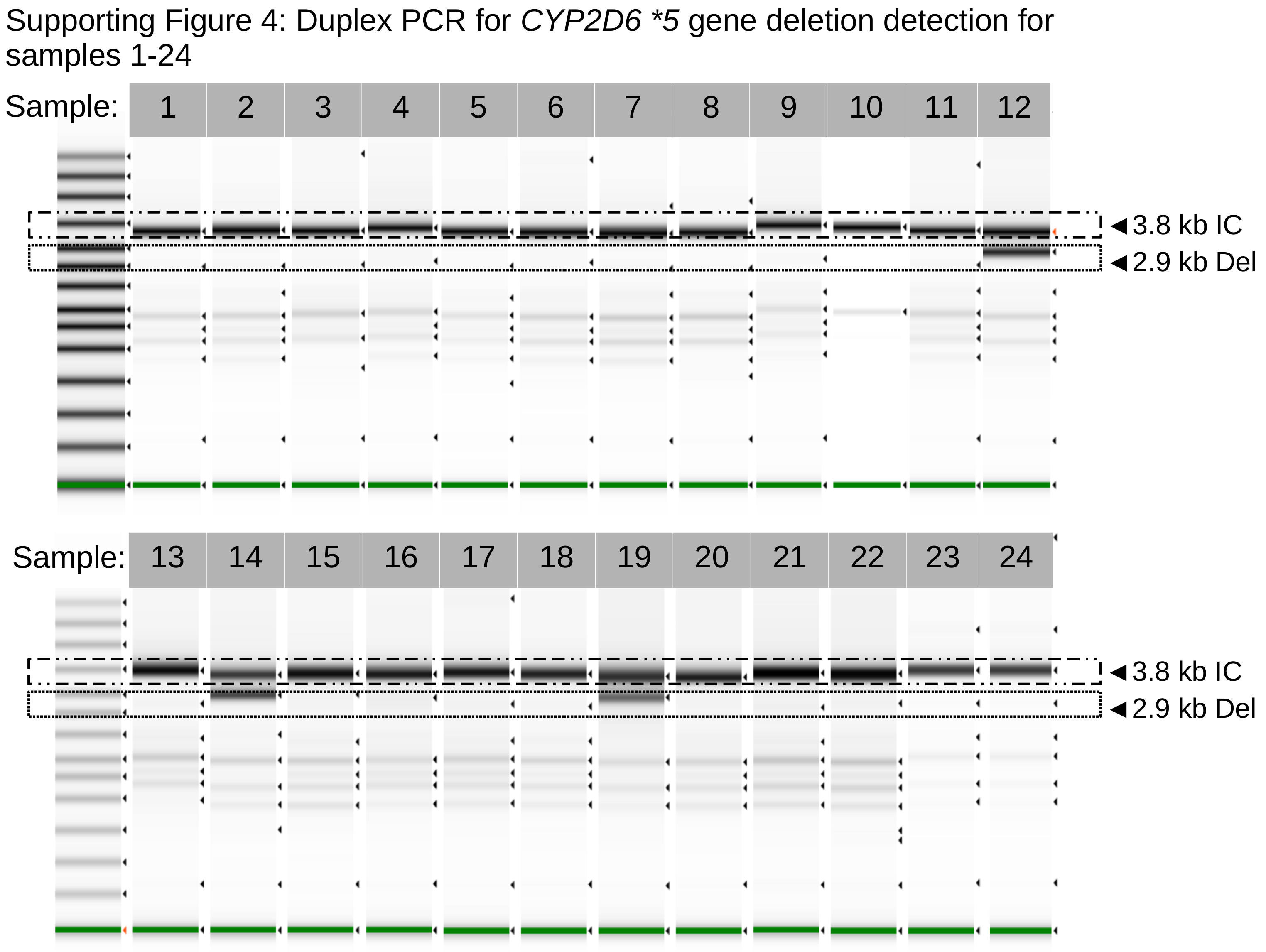
Supporting Figure S4: CYP2D6 duplex PCR assays

Duplex PCR results for all 24 samples. The 3.8 kb fragment A serves as an internal control and should be present in all reactions. Presence of the 2.9 kb fragment is indicative of a *CYP2D6* gene deletion (*CYP2D6 *5*; samples 12, 14,19).
